# Sleep in older adults

**DOI:** 10.1093/geroni/igaf122.1913

**Published:** 2025-12-31

**Authors:** Glenna Brewster

**Affiliations:** Emory University, Atlanta, Georgia, United States

## Abstract

This session shares research on the association and impact of sleep in older adults.

